# Graphene/fluorescein dye-based sensor for detecting As(III) in drinking water

**DOI:** 10.1038/s41598-021-96968-3

**Published:** 2021-08-27

**Authors:** Madhu D. Sharma, Sadhana S. Rayalu, Spas D. Kolev, Reddithota J. Krupadam

**Affiliations:** 1 Environmental Materials Division, CSIR-National Environmental Engineering Research Institute, Nehru Marg, Nagpur, 440020 India; 2 School of Chemistry, The University of Melbourne, Parkville, VIC 3010 Australia

**Keywords:** Fluorescent probes, Optical properties and devices

## Abstract

A complex of reduced graphene oxide (rGO) and fluorescein (FL) dye nanoparticles of size between 50 and 100 nm has been prepared and its sensing performance for detection of As(III) in drinking water has been reported. When As(III) binds to the rGO–FL nanoparticles the relative quenching of fluorescence was increased with increase in As(III) concentration thus provide two linear calibration ranges (0–4.0 mmol L^−1^ and 4.0–10 mmol L^−1^). The fluorescence quenching mechanism was investigated by using time-resolved fluorescence spectroscopy and molecular modeling. The detection limit of this sensor has been determined as equal to 0.96 µg L^−1^ which is about 10 times lower than the WHO stipulated standard for As(III) in drinking water (10 µg L^−1^). The analytical performance and potential application of the nanosensor was compared to commercial field kits used in arsenic monitoring. The sensor proposed in this study is fast, sensitive and accurate for detection of As(III) in drinking water and environmental samples.

## Introduction

Contamination of drinking water with toxic arsenic species has been reported across the globe^1,2^. The inorganic forms of arsenic exhibit higher toxicity as compared to the organic forms^3^. The standard prescribed for arsenic in drinking water by World Health Organization (WHO)^4^ is 10 µg L^−1^. The testing of drinking water for arsenic species such low concentration is a challenge to analysts. Sophisticated instrumental techniques such as inductively coupled plasma mass spectrometry (ICP-MS) are often used to determine ultra-trace concentrations of arsenic species in water^5^. This method is expensive and not fit for *in-situ* analysis. Indeed, there is a great demand for in-situ, portable, and sensitive methods or devices for As(III) detection in drinking water and environmental samples.

Recent advancements in the fields of nanoscience, colloids and interfaces have been useful in the development of highly sensitive sensors for the detection of environmental pollutants such as endocrine disrupting chemicals (EDCs), carcinogens, pesticides, explosives, food toxins and other toxic chemicals^6^. The wonder material of twenty-first century is graphene which has great potential in sensor technology for detecting gases, chemicals, heavy metal ions, and other environmental pollutants. The current advances, sensitivity and selectivity of a wide variety of graphene-based sensors have been reported^7,8^. Ultra-sensitive detection of As(III) in the concentration range of 1.0–10 nmol L^−1^ was reported using a graphene-lead oxide electrode^9^. The use of lead oxide may be instrumental in improving the sensing performance of this electrode, but it is a highly toxic compound. An aptamer based fluorimetric method has offered an impressively low detection limit of 1.3 pmol L^−1^ for As(III). In this method, the highly toxic cadmium ion was used as the aptamer conjugate to generate fluorescence signal. Another limitation of this method stems from the fact that the aptamer can be easily denatured^10^. A wide detection concentration range (1–500 µg L^−1^) of As(III) was achieved by using a Au-based nanoparticle sensor, however, the cost of measurement would be expensive because of Au was used in the sensor fabrication^11^. Zeng et al. reported an aptamer formulated with DNAzyme for As(III) detection in the picomolar concentration range^12^. A renewable gold plated Ir-based microelectrode was developed for the detection of As(III) at concentrations between 10 and 50 nmol L^−1^ at pH 8.0^13^. A biosensor based on Au@Ag core–shell nanoparticles for SERS detection of As(III)^14^ has exhibited a detection limit of 0.1 μg L^−1^. A nanoprobe was fabricated with the combination of aptamers and mesoporous silica nanoparticles which showed highly sensitive detection of As(III) in aqueous solutions^15^.

Ezeh and Harrop was formulated a new reagent named as “ArsenoFluor1 (AF1)” to determine As(III) in organic solvents at 298 K^16^. This reagent emits strong fluorescence emission at 496 nm which is specific for As(III). The media used in this method for As(III) analysis are organic solutions which limits application of this method for water monitoring. A rapid and sensitive detection of As(III) using fluorescent test papers embedded with quantum dots was reported^17^. The fluorescent paper turns from red to cyan in the presence of As(III) in aqueous solutions; and this method can detect As(III) as low as 2.0 µg L^−1^. Steinmaus et al. evaluated the performance of two field kits for arsenic analysis (Quick Arsenic and Hach EZ) and as compared their sensing performance with atomic fluorescence spectrometric method^18^. Both kits have shown As(III) detection in the range of 15–20 µg L^−1^ which is above the WHO stipulated standard for drinking water of 10 µg L^−1^. A list of sensor/methods for detection of As(III) in environmental samples at concentrations lower than 10 µg L^−1^ is given in Table 1.

**Table 1:** Highly sensitive arsenic detection/sensing systems reported in the literature.

Sr. no	Sensing/detection system	Sensitivity/lower detection limit	References
1	GO-PbO composite	0.01 ppb	^9^
2	CdTe/ZnS core/shell QDs using Aptamer	< 1 ppb	^10^
3	AuNPs nanoparticle-based biosensor	40 ppb for naked eye 0.6 ppb for colorimetric assay 0.77 ppb for RS assay	^11^
4	Aptasensor with DNAzyme canalytic amplifier	10 pM	^12^
5	Gold plated Ir-based microelectrode (AuCs)	< 1 ppb	^13^
6	Surface plasmon resonance (SPR) sensor using several thiol-containing organic compounds	10 ppb	^14^
7	Luminescent Bacterial Biosensor	< 1 ppb	^15^
8	ArsenoFluor1 fluorescent chemical probe	10 ppb	^16^
9	Modified Quantum dots (QDs) mixed with cyan carbon dots (CDs)	1.7 ppb	^17^
10	rFO-FL nanosensor	1.0 ppb	In this study

**Figure 1:**
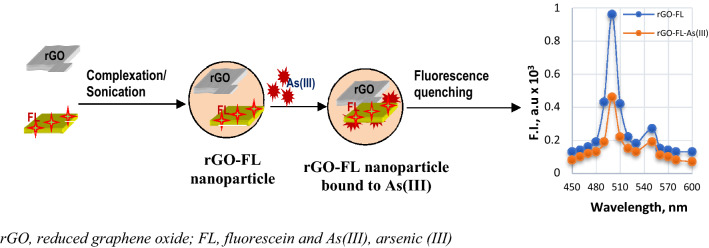
The sensing schematic of rGO–FL nanosensor for As (III) detection.

In this study, we report the reduced graphene oxide (rGO) and fluorescein (FL) dye nanocomposite-based As(III) sensor. The sensing schematic of rGO–FL nanosensor for As(III) detection is given in Fig. 1. The rGO/FL nanocomposite was characterized by using scanning electron microscope (SEM), atomic force microscope (AFM), X-ray photoluminescence spectrometer (XPS), infrared spectrometer (IR), dynamic light scattering (DLS) system and Zetanano sizer (ZNS) and fluorescence spectrophotometer (FS). The rGO/FL nanosensor respond to As(III) in aqueous solutions by quenching fluorescence emission. The mechanism of fluorescence quenching was studied using time-resolved fluorescence spectrophotometry. The stability of the rGO/FL nanosensor in aqueous solution was investigated by using a dynamic light scattering method. The nature of interactions between the colloidal particles and As(III) was modeled using Gaussian Ver 4.21 software. The rGO/FL nanosensor showed lower limit of detection of 0.96 μg L^−1^ for As(III) in drinking water, much better than that of WHO recommended standard of 10 μg L^−1^. The method developed for As(III) detection in this study was compared to the standard APHA Standard Method based on the use of ICP-OES/MS and portable As(III) kits/devices used in in-situ monitoring of arsenic in drinking water and environmental samples.

## Methods

### Materials

Graphene was prepared from graphite flakes (purchased from Sigma-Aldrich, St. Louis, United States) by the Hummers method^19^. Graphene was chemically exfoliated to produce graphene oxide (GO)^20^. The GO was reduced by using hydrazine hydrate to obtain reduced graphene oxide (rGO)^21^. Fluorescein dye (FL, SKU-46955) and a certified reference material (TraceCERT-71718) of As(III) were purchased from Sigma Aldrich (St. Louis, United States). The concentration of As(III) this standard solution was 1.0 ± 0.01 mg L^−1^. Reagent grade water of quality Type I (ASTM D1193-91) was used during preparation of the rGO–FL nanoparicle solution. Reagent grade purity of water with conductivity lower than 0.01 µS cm^−1^ and total organic carbon lower than 20 µg L^−1^ was in the preparation of solutions. The pH of the reagent-grade water was 5.83 ± 0.05. The other chemicals used in this study were procured from Merck (Kenilworth, USA) and used as procured.

### Preparation of graphene-dye nanosensor

**Figure 2:**
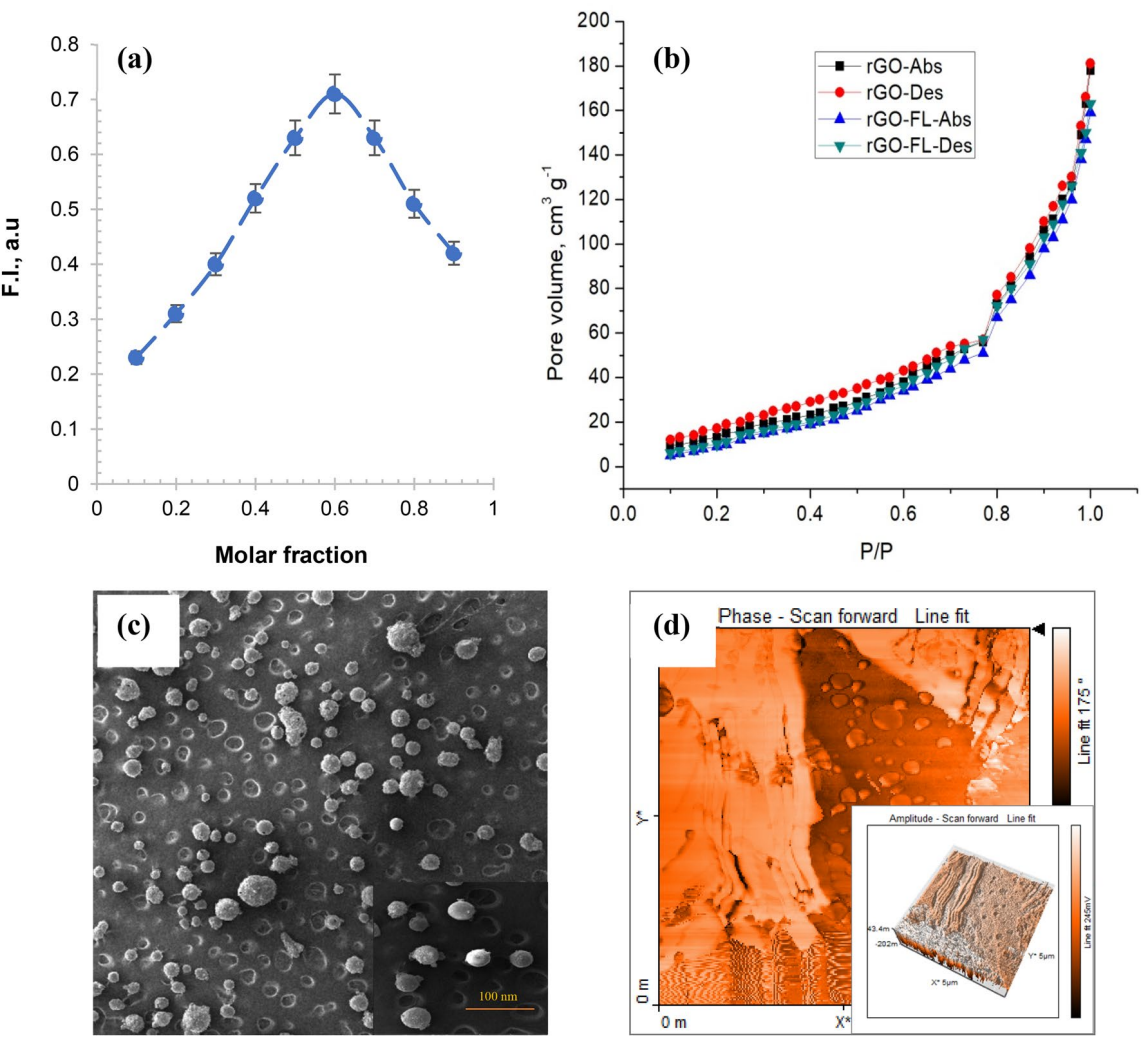
(**a**) Job’s plot for optimum stoichiometric complex formation between rGO and FL. Job’s plot provides qualitative and quantitative details about the stoichiometry of association between reduced graphene oxide (rGO) and ligand (Fluorescenc) to form rGO–FL complex contain 0.7 mL of 1 mmol rGO and 1 mmol of fluorescein. The complex formation between rGO and FL was studied using fluorescence emission at 510 nm (**b**) Nitrogen adsorption–desorption isotherms of rGO and rGO–FL particles (**c**) SEM micrograph (**d**) AFM micrographs of rGO–FL nanoparticles.

Initially, the stoichiometric composition of rGO–FL nanoparticles was determined by using Job’s plot^22^. The optimum ratio of rGO and FL was determined by monitoring the fluorescence emission at different mole fractions. The rGO–FL nanoparticles with optimal composition were used to detect As(III) in water by measuring the change in the intensity of fluorescence quenching. It was found that the fluorescence emission at 510 nm showed the highest intensity for rGO–FL nanoparticles with a ratio of 6:4 (Fig. 2a). The rGO–FL nanoparticles were prepared by sonicating 6 g of rGO in 100 mL of reagent grade water for 10 min, followed by addition of 4 g of fluorescein. The nanparticles were further sonicated for 10 min. After keeping the suspension for 1 h, the solution was filtered through Whatmann filter paper No. 41 (pore size, 20 μm). The fine particles in the filtrate were separated by centrifugation. The particles collected on the filter paper and particles collected from centrifugation were washed thoroughly with 100 mL of reagent-grade water. The washing was repeated 5 times and then the nanoparticles were dried in vacuum before use as the nanosensor for detection of As(III) in water.

### Instruments

The surface area, pore volume and average pore diameter of rGO–FL nanoparticles were determined by using N_2_ adsorption isotherms obtained from an Accelerated Surface Area and Porosimetry System (Micromeritics-ASAP 2420, USA) at 77 K. Nitrogen adsorption surface area of the nanoparticles was computed by Brunauer–Emmett–Teller (BET) equation^23^ and pore volumes were calculated using Barret–Joyner–Haldena (BJH) method^24^. Microscopic observation of the nanoparticles was carried out using a scanning electron microscope (SEM) (S-2500 Hitachi, Japan). The surface morphology of the rGO–FL nanoparticles was viewed by using an atomic force microscope (AFM) (FlexAFM, Nanosurf AG, Switzerland). The micrographs of the rGO–FL nanoparticles were obtained at image pixel size 2.5 µm. The zeta potential of nanoparticles was measured using a two-angle particle and molecular size analyzer (Zetasizer Nano ZS, Malvern Pananalytical, United Kingdom). The infrared spectra of the rGO, rGO–FL nanoparticles and rGO–FL nanoparticles with As(III) were obtained by an infrared spectrometer (Perkin-Elmer 983 G, USA). The fluorescence emission at 510 nm was measured by a spectrofluorimeter (Hitachi F-7500, Tokyo, Japan) by exciting samples at 348 nm. The dynamic light scattering (DLS) method was used to measure the particle size of the rGO–FL nanoparticles in water using a Nano size Analyzer (Horiba-SZ 100, Japan). This analyzer is equipped with a laser beam at 532 nm. X-ray photoelectron spectroscopy (XPS) measurements of samples were performed on a spectrometer (Scienta Omicron, Germany) hyphenated with an electron spectroscopy for chemical analysis (ESCA) probe using Al-K_ἀ_ radiation source to generate monochromatic beam with energy *hν* = 1486.6 eV.

### Molecular modeling

The molecular model of the rGO–FL nanoparticles with conformational structure was developed on HyperChem Rel 8.0 software. The probable sites for As(III) binding were identified by computing the intrinsic molecular energy in a geometrically optimized rGO–FL nanoparticle^25^. The force fields module MM2 in the software Gaussian Ver 4.21 was used to identify the probable sites with high affinity for As(III) in water^26^.

### Experimental procedure

A known quantity (10 mg) of the rGO–FL nanoparticles was added to 10 mL of reagent grade water and the fluorescence emission at 510 nm was measured using a fluorescence spectrometer. Similarly, the same amount of the rGO–FL nanoparticles was added to different volumes of reagent water and the fluorescence emissions were recorded. The agglomeration of nanoparticles in water was prevented by adding a few drops of Caster Oil (CO) during the As(III) measurement. The kinetics of aggregation was monitored by recording fluorescence quenching as a function of time.

The fluorescence emission intensity values of the rGO–FL nanoparticles (F_o_) and the rGO–FL-As(III) complex (F_I_) after the addition of different concentrations of As(III) were recorded during calibration. The pH effect on As(III) sensing was determined by varying pH of the As(III) solutions with the addition of 0.01 M HNO_3_ or 0.01 M NaOH solutions. By spiking the As(III) solution with certified reference pH buffers 4.75, 7.4 and 9.25, the fluorescence emission was recorded. The cross-selectivity experiments were performed as follows: the solution of metal ions containing all the metal ions of interest was prepared by dissolving the relevant metal salts in deionized water. The initial precipitate of metal hydroxides was dissolved by adding a few milliliters of HNO_3_ to achieve a final pH of the solution between 1.8 and 2.1. The concentration of each metal ion in the solution was equal to 1.0 µg L^−1^. A given amount of the rGO–FL nanoparticles was added to the metal solution and the mixture was stirred by a magnetic stirrer at room temperature. Then, the residual concentration of each metal ion at predetermined time intervals was measured by ICP-OES. The quantity of each metal ion uptake per gram of rGO–FL nanoparticles was determined by the difference between the initial and final concentrations of the metal ion in the test solutions.

### Interference of sample matrix parameters

The two important sample matrix parameters—otal dissolved solids (TDS) and dissolved organic matter (DOM)—which interfere during the analysis of As(III) in water samples. The interference experiments were conducted as follows: the groundwater samples collected from different locations with varied concentrations of TDS and DOC and to each sample a standard As(III) solution of concentration 1.0 µg L^-1^ was added. The samples with different TDS and DOC concentration were added 10 mg of the rGO–FL nanoparticles. Humic acid (HA) was used as a representative chemical of organic matter in sample solutions. A certain quantity of HA (10–500 µg L^−1^) was added to As(III) standard solutions to examine its effect on detection of As(III) by the rGO–FL nanosensor. The groundwater samples and samples spiked with a standard As(III) solution were simultaneously analyzed and the analytical sensitivity and limits of detection of the newly developed rGO/FL sensor was determined. The rGO–FL sensor performance was compared to field kits and the Standard Method (APHA, 2017; Method, 3120)^5^.

## Results and discussion

### Properties of the rGO/FL nanosensor

**Figure 3:**
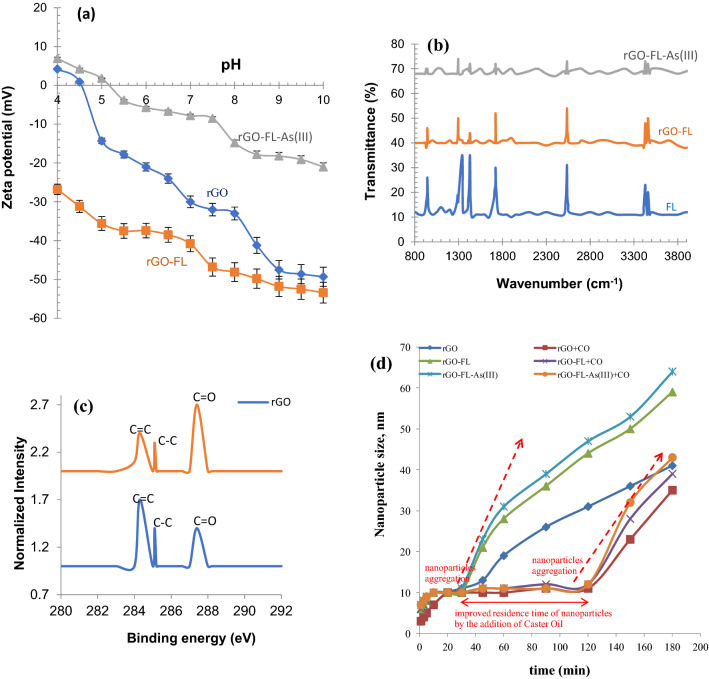
(**a**) Zeta-potential of rGO, rGO–FL and rGO–FL-As(III) nanoparticles (**b**) Infrared spectra of FL, rGO–FL and rGO–FL complex with As(III) (**c**) XPS spectra of rGO and rGO–FL (**d**) Dynamic light scattering results illustrating the growth of the rGO–FL nanoparticles as a function of time.

The nanoparticles were spherical in shape with the size varied between 50 and 100 nm. The particles of size < 50 nm was about 65% and the average particle diameter of rGO/FL nanoparticles was 28.9 nm. The specific surface area of rGO–FL nanoparticles was 164 m^2^ g^−1^. The surface area of rGO without FL was 182 m^2^ g^−1^ and higher surface area was attributed to the higher hydrophilicity of the rGO–FL nanoparticles which facilitated their aggregation in water. The N_2_ adsorption–desorption curves followed type IV model^27^ with a weak hysteresis loop in the relative pressure range 0.5–1.0 (Fig. 2b). The SEM micrographs of rGO–FL nanoparticles showed the existence of micro/meso-pores (Fig. 2c). The topology of rGO/FL nanoparticles were analyzed with high resolution AFM. The micrographs of AFM showed the scattered cavities with a depth of 5 ± 2 nm. It can be hypothesized that these cavities could be preferentially binding with As(III) species (Fig. 2d). The AFM micrographs of 3D views further confirm the existence of cavities on the surface of rGO–FL nanoparticles. The zeta potential of the rGO nanoparticles decreased with an increase in pH and reached isoelectric point at pH 4.48 (Fig. 3a). When the rGO nanoparticles were complexed with FL, the isoelectric point was shifted upward from 4.48 to 5.27. The rGO–FL nanoparticles were reported to exhibit high stability between pH 6 to pH 8 where the zeta potential was below − 30 mV. The rGO/FL nanoparticles improved the dispersibility and stability of the particles due to their mutual repulsion in water^28^. The infrared spectra of rGO–FL nanoparticles show the stretching vibration peaks at 3460 (–C=O–), 3429 cm^−1^ (–O–H), 1721 cm^−1^ (C=O–H), 1429 (C–O), and 1296 cm^−1^ (–C–H) indicating the presence of fluorescein functionalities on the surface of the rGO particles. These peaks were reduced in intensity or disappeared after exposure of the particles to As(III) (Fig. 3b). The XPS measurements showed a weak peak of O^1^ s for the rGO–FL nanoparticles thus indicating low residual oxygen functionalities (Fig. 3c). The C^1^*s* XPS spectra of the rGO and rGO–FL nanoparticles showed the peaks specific to carbon functionalities at 284.3 eV (C in C=C bond), 285.1 eV (C in C–C bond) and 287.4 eV (C in C=O bond)^29^. The intensity of the peak at 283.3 eV was reduced significantly in rGO–FL nanoparticles due to shifting of *sp*^2^ hybridization of C=C to *sp*^3^ hybridization of C–C. This XPS data provide a proof of the existence of more C=O functionalities in the rGO–FL nanoparticles attribute to the formation of a rGO/FL complex. The DLS results have shown that the nanoparticles interact with each other and after 45 min the particles start to agglomerate in water. To prevent this agglomeration, 10 mL of Caster Oil (CO) was used in 1.0 L of rGO–FL nanoparticle solution. The addition of CO prolonged the particle agglomeration time from 45 to 120 min (Fig. 3d). The maximum particle size, measured by using DLS, ranged from 25 to 57 nm. The DLS particle size distribution data indicate that 20% increase in the size of the rGO–FL complexed with As(III) compared to solitary rGO–FL nanoparticles. An increase in the size of nanoparticles facilitates the agglomeration^30^.

### Interaction of the rGO–FL complex with As(III)

**Figure 4:**
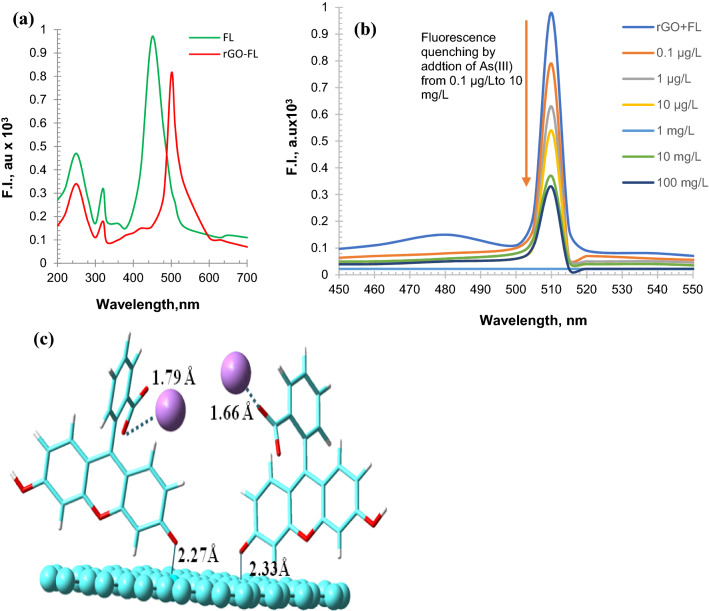
(**a**) Fluorescence emission spectra of FL and rGO–FL showing red shift during formation of nanocomposite (**b**) Fluorescence titration spectra of rGO and FL in aqueous solution. The fluorescence emission was observed at 510 nm when the sample was excited at 348 nm and the increase in concentration of As(III) reduce the fluorescence intensity (**c**) Molecular model of rGO–FL nanoparticles interaction with As(III) simulated in vacuum using Gaussian Ver. 4.2.1 software; https://gaussian.com/utils.

When the rGO–FL nanoparticles interact with As(III) the fluorescence emission was quenched. This quenching phenomenon was characterized by (1) a red shift of the fluorescence emission of rGO from 453 to 510 nm when rGO was complexed with FL (Fig. 4a) and (2) the quenching of the fluorescence of the rGO–FL particles upon binding with As(III). The fluorescent titration of rGO–FL nanoparticles with As(III) have shown that the intensity of fluorescence emission at 510 nm decreased when the concentration of As(III) increased from 0.1 µg L^−1^ to 100 mg L^−1^ (Fig. 4b). The fluorescence quenching would be attributed to the binding of As(III) on to the surface functional groups of the rGO–FL nanoparticles. The binding of carboxylic acid and ketonic groups of FL to the rGO nanoparticles red shifted in the fluorescence emission. The electronegativity of the rGO–FL nanoparticles was also increased. Furthermore, molecular modeling showed the rGO–FL nanoparticles interact with arsenic in the neutral arsenious acid (H_3_AsO_3_) form which is dominant at pH between 5 and 7 in natural waters. This interaction facilitate transfer of energy between rGO–FL nanoparticles and As(III) thus reducing the distance between them from 2.33 to 2.27 Å (Fig. 4c). The rGO–FL nanoparticles form a flexible bi-layer structure in which the –COOH, –OH and =O functionalities of FL are bound to the –CONH_2_ and =C=O– groups of rGO. These model findings were consistent with the infrared spectra of the rGO–FL particles prior and after complexation with As(III) (Fig. 3b). The graphene performs two important functions. They are (1) providing high surface area (164 m^2^ g^−1^) substrate for hosting fluorescein dye and this dye interacts with As(III) species in the aqueous solution and (2) the graphene interaction with FL dye red shift the fluorescence signal which is represents stabilized and quantifiable fluorescence quenching as shown in Fig. 4a.

Based on the Job’s plot, the rGO and FL ratio in the nanoparticles was found to be 6:4 and the formation of rGO–FL complex was evidenced by the following experimental results: (1) the isoelectric point of rGO was shifted from pH 4.48 to pH 5.27 after the formation of rGO–FL complex (2) presence of infrared peaks at 3460 (–C=O–), 3429 cm^−1^ (–O–H), 1721 cm^−1^ (C=O–H), 1429 (C–O), and 1296 cm^−1^ (–C–H) reflecting the fluorescein functionalities in the rGO–FL complex and (3) the XPS peak at 283.3 eV was reduced significantly in rGO–FL particles due to change of *sp*^2^ to *sp*^3^ hybridization of C–C indicating more C=O functionalities^31^. Furthermore, bonding between rGO and FL was established by the molecular model which is complemented with fluorescence red shift, i.e., shift from 453 to 510 nm when the rGO forms a complex with FL.

### Effect of pH

**Figure 5:**
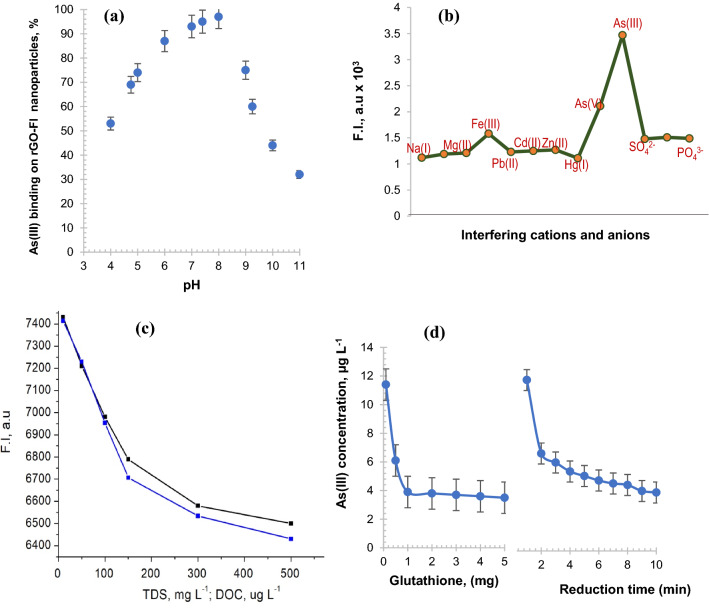
(**a**) Effect of pH on the fluorescence emission of rGO–FL nanoparticles (**b**) Interference of metal ions and common anion during detection of As(III) by the rGO–FL nanoparticle sensor (**c**) Interference of total dissolved solids (TDS) and dissolved organic carbon (DOC) during As(III) detection by using rGO–FL nanoparticle sensor (**d**) quantity of glutathione required for reduction of As(V) to As(III) and the time of reduction.

The fluorescence quenching of a solution containing 0.1 μg L^−1^ As(III) and 100 mg L^−1^ rGO–FL nanoparticles increased with pH between 3.0 to 8.0 when As(III) was present in the form of the neutral arsenious acid (H_3_AsO_3_) and then declined further with increase in pH from 8.0 to 10.0 (Fig. 5a). The decline could be explained by the deprotonation of H_3_AsO_3_ resulting in the negatively charged H_2_AsO_3_^−^ anion being repelled by the negatively charged nanoparticles. At pH < 8.0, the size of the nanoparticles was small (20 nm) and increased to 80 nm at pH 10.0. When the nanoparticle increases in size it would be repelling anions e.g., sulphate, nitrate and phosphate^32^. The change in pH has no significant effect on the quantum confinement of the rGO–FL particles particularly in the pH range 6.5–8.5 which is the range practically required for drinking water quality testing.

### Selectivity of the nanosensor

The effect of other metal ions during detection of As(III) in water was investigated by using a competitive binding assay. The rGO–FL nanoparticles selectively bind the target As(III) at the concentration of 1.0 µg L^−1^ in the presence of other metal ions (of concentration 1.0 µg L^−1^ of each metal ion) and As(V). The experimental data, presented in Fig. 5b, indicate that none of the metal ions have a significant effect on the selective sensing of As(III) in water. The slight reduction in sensing of As(III) was found in the co-existence of anions and cations may be attributed to competition for adsorption sites between the co-existing ions and arsenic species.The As(III) species have less electronegativity (1.549) compared with As(V) species (2.40) and other metals. The zeta potential measurement for net negative charge of the rGO–FL nanoparticles further confirmed their preferable binding to As(III) species in water (Fig. 3a). This statement was furthermore confirmed by the interference of commonly found anions in water i.e., sulphate, nitrate and phosphate. The interference of these anions was higher than the other metal ions. There is another approach based on quantum confinement which is size dependent phenomena^33^. The rGO nanoparticles containing FL cause intrinsic fluorescence emission due to conjugated π-domains. The anionic species (sulphate, nitrate and phosphate) are potential interferants and therefore it was found that these anions have shown higher interference compared to the metal cations.

### Effect of sample matrix parameters

To test the practical application of the rGO–FL nanoparticles as sensitive sensors, we used groundwater samples collected from different parts of India and then samples were spiked with standard As(III) of concentration 0.1 μg L^−1^. The sample matrix parameters, total dissolved solids (TDS) and dissolved organic carbon (DOC), were determined by using the Standard Methods^5^. It was found that the groundwater samples with TDS value above 1500 mg L^−1^ interfere in As(III) detection significantly. The interference of DOC was more pronounced than the TDS. At 250 µg L^−1^ of DOC in the water sample, the fluorescence emission was reduced to 40%. Figure 5c show the effect of DOC on sensing performance of rGO–FL nanoparticles. The nanoparticle sensor lost its sensing below 50% when the DOC concentration was reached to 200 µg L^−1^. This may be due to fast and strong bond formation between the organic moieties of HA with As(III) as compared to TDS with the rGO–FL nanoparticles. The interference of TDS and DOC during detection of As(III) in water by the proposed sensing material is quite significant. The boundary concentration of TDS and DOC were 1500 mg L^−1^ and 250 μ L^−1^ for water sample. The sample pre-treatment would be proposed to improve the detection of As(II) in water, when the samples were collected from natural resources with high TDS and DOC concentration.

### Sample preparation for As(III)

Since As(V) species are dominant in natural waters and it is necessary to reduce As(V) to As(III) for improving the detection of the rGO–FL nanoparticles. As (III) is highly toxic as compared to As (V). Even though, natural water contains As (V) as the dominant species, the existence of As (III) is reported as a minor fraction. Indeed, the groundwater resources where anoxic conditions prevail, the As(III) is the dominant arsenic species. Many Asian regions depend on groundwater resources for drinking purpose. With this context, the proposed sensor has importance to detect traces of As(III) species in the public health point of view. Glutathione was used in this study to reduce As(V) to As(III). The concentrations of the reductant and time for reduction were optimized. The quantity of glutathione required for 100 mL of water sample was 6.3 mg and the time required for reduction was 2 min when the concentration of As(V) was 10 mg L^−1^ (Fig. 5d). In this study high concentration of As(III) solution was chosen since it was approximately 10 times higher than the concentration of As(III) in groundwater samples which were the realistic concentration in Bengal region in India. The quality control/assurance of experiments were conducted as per the Standard Methods (APHA, 2017; 23rd ds.)^5^.

### Analytical figures of merits

**Figure 6:**
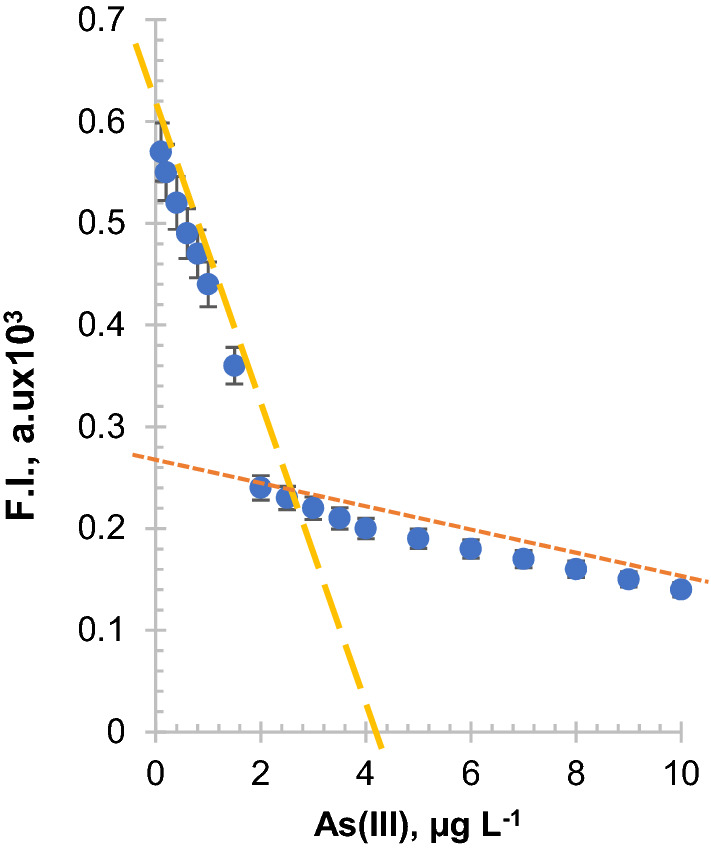
The Stern–Volmer plot of the quenching of the fluorescence emission of rGO–FL nanoparticles during adsorption of As(III) in aqueous solutions.

The Stern–Volmer plot of rGO–FL fluorescence emission versus the concentration of As(III) have shown the typical trend of fluorescence quenching (Fig. 6). A linear plot with a slope of 0.98 × 10^–7^ M was obtained. This slope indicates the formation of a stable complex between the rGO–FL and As(III). The limit of detection was calculated from the ordinate intercept of linear regression between the fluorescence intensity of rGO–FL nanoparticles versus concentration of As(III). A signal-to-noise ratio is calculated based on fluorescence peak height quenched during the addition of standard As(III) solution and measured from extrapolated baseline signal equal to half-height of the peak-to-leak background noise. The detection limit was computed from the relationship pf 3S_B_/S. The limit of minimum quantification of As(III) could be detected by the rGO–FL nanoparticles was 0.098 μg L^−1^; and this lower detection limit is about 10 times better than the WHO guidelines i.e., 10 μg L^−1^.

The analytical performance of the rGO–FL nanosensor was compared with the Standard Method (APHA, 2017: Method 3120) and two different types of field kits (Quick Arsenic and Hach EZ) and *in-situ* devices (Wagtech Digital and Chem-In Corp)^34,35^. The detection limit for As(III) of the methods mentioned above varied between 0.1 µg L^−1^ and 10 mg L^−1^. The rGO–FL nanoparticles can be added to the sample without any sample pretreatment followed by directly monitoring the fluorescence signal. The detection limits of the method is quite suitable for real samples analysis and the value is about 10 times higher sensitivity as compared to the WHO standard for drinking water i.e., 10 μg L^−1^. Unlike the APHA standard method, the proposed method does not require advanced instrumentation (ICP-MS), high energy demand and skilled manpower. The measurement of uncertainty of the proposed method is quite low compared with the standard method and the kits/devices^36^. Even though, the field kits and devices are very comfortable for *in-situ* monitoring, due to their lower sensitivity for As(III) and high cost prevents wide field applications. The summary of analytical figures of merits of the newly developed sensor and other methods were given in Table 2. Overall, the new sensor proposed in this study is inexpensive, simple to prepare and use, environment-friendly and sensitive enough to meet the regulatory standard for As(III) analysis in environmental and drinking water.

**Table 2:** Comparison of performance and cost of As(III) determination using various recently reported methods/sensors and field kits.

Method/sensor/kit	Limits of detection, mg L^−1^	MU*	LOD, µg L^−1^	Approx. Cost, USD	Remarks	References
ICP-OES	0.5–100	± 0.2	50	6–8	Interference of other metals Expensive Skilled manpower and advanced equipment	^6^
ICP-MS	0.001–100	± 0.01	1	8–10	Expensive Advanced equipment Skilled manpower	^6^
Hach EZ kit	15–50	± 1.5	150	4–5	Do not meet WHO Standard for drinking water, 10 µg L^−1^	^18^
Quick Arsenic	12–50	± 0.9	200	4–5	Do not meet WHO Standard for drinking water, 10 µg L^−1^	^18^
Wagtech Digital	18–40	± 1.1	200	3–4	Do not meet WHO Standard for drinking water, 10 µg L^−1^	^26^
Chem-In Corp	20–50	± 1.4	150	3–4	Do not meet WHO Standard for drinking water, 10 µg L^−1^	^34^
rGO–FL nanosensor	0.001–10	± 0.04	1.0	1–2	Highly sensitive and meet the WHO drinking water quality standard Other metals interference is negligible Reusable sensing materials	In this study

*MU, measurement of uncertainty was calculated according to the procedure given in Ref.^13^. The number of samples which were tested was 5. Out of 5 samples, 2 samples were reagent water spiked with As(III) standard of different concentrations and remaining 3 samples collected from different locations in India. Each sample was tested repeatedly for seven times (n = 7).

### Analysis of environmental samples

To demonstrate the feasibility of the rGO–FL nanosensor, three real groundwater samples were collected from different parts of India and were analyzed. Table 3 lists the concentration of As(III) found in real samples which varied between 1.0 and 20 µg L^−1^. The accuracy of the sensor performance was evaluated by a recovery test using spiked groundwater samples with two different concentrations, i.e., 1.0 and 10 µg L^−1^. The recoveries ranged between 99 and 101% thus indicating satisfactory accuracy.

**Table 3:** Determination of As(III) in original and spiked groundwater samples and a standard reference material (TraceCERT®-71718 with the concentration of 10 mg L^−1^ 0using rGO–FL nanoparticle based fluorescence sensor.

Sample	As(III) added, µg L^−1^	As(III) determined, µg L^−1^	Recovery, %	Remarks
G-1	0	4.08 ± 0.12	N/A0	TDS, 210 mg L^−1^ DOC, 60 µg L^−1^; pH, 6.9
	0.1	4.14 ± 0.27	99.7	
	10	14.15 ± 0.25	100.3	
G-2	0	5.98 ± 0.15	N/A	TDS, 550 mg L^−1^; DOC, 90 µg L^−1^; pH, 7.5
	0.1	6.12 ± 0.24	101.8	
	10	15.99 ± 0.33	99.8	
G-3	0	3.11 ± 0.1	N/A	TDS, 2100 mg L^−1^; DOC, 250 µg L^−1^, pH, 7.2
	0.1	3.15 ± 0.21	100.4	
	10	13.21 ± 0.27	101.1	
As(III) Std. TraceCERT(R)_ 72718	0.1	0.97 ± 0.13	99.7	TDS, 20 mg L^−1^; DOC, < 10 µg L^−1^; pH, 5.9
	10	9.98 ± 0.11	99.3	

*G-1* Groundwater sample was collected from the rural area near Nagpur, Maharashtra, India, *G-2* the groundwater sample was collected from urban area near Nagpur, Maharashtra, India, *G-3* the sample was collected near coastal area at Bhadbhut, Gujarat, India, *TDS* total dissolved solids, *DOC* dissolved organic carbon.

## Conclusions

The graphene-dye nanosensor is useful to detect ultra-trace concentrations of As(III) in drinking water and environmental samples and the proposed detection is sensitive compared to the Standard Methods (APHA, 2017) and commercially used field kits/devices. The two important features of this sensor responsible for such high sensitivity. They are (1) the rGO–FL particles produce very strong and distinct fluorescence emission at 510 nm and (2) interaction of As(III) with the rGO–FL nanoparticles is specific and linearly quenches fluorescence emission with an increase in the As(III) concentration in the water samples/standards in water samples. The time-resolved fluorescence investigation suggested that the nanoparticle agglomeration could be prevented up to 120 min by adding traces of castor oil. In case of high As(V) concentration in the water samples, a simple natural glutathione reductant would be sufficient to convert As(V) to As(II) within 10 min. The proposed new sensor is highly sensitive and cost-effective for monitoring of As(III) in drinking water and environmental samples.
